# Cloning and Characterization of Genes Involved in Nostoxanthin Biosynthesis of *Sphingomonas elodea* ATCC 31461

**DOI:** 10.1371/journal.pone.0035099

**Published:** 2012-04-11

**Authors:** Liang Zhu, Xuechang Wu, Ou Li, Chaodong Qian, Haichun Gao

**Affiliations:** Institute of Microbiology, College of Life Sciences, Zhejiang University, Hangzhou, Zhejiang, People's Republic of China; University of Wisconsin, Food Research Institute, United States of America

## Abstract

Most *Sphingomonas* species synthesize the yellow carotenoid nostoxanthin. However, the carotenoid biosynthetic pathway of these species remains unclear. In this study, we cloned and characterized a carotenoid biosynthesis gene cluster containing four carotenogenic genes (*crtG*, *crtY*, *crtI* and *crtB*) and a β-carotene hydroxylase gene (*crtZ*) located outside the cluster, from the gellan-gum producing bacterium *Sphingomonas elodea* ATCC 31461. Each of these genes was inactivated, and the biochemical function of each gene was confirmed based on chromatographic and spectroscopic analysis of the intermediates accumulated in the knockout mutants. Moreover, the *crtG* gene encoding the 2,2′-β-hydroxylase and the *crtZ* gene encoding the β-carotene hydroxylase, both responsible for hydroxylation of β-carotene, were confirmed by complementation studies using *Escherichia coli* producing different carotenoids. Expression of *crtG* in zeaxanthin and β-carotene accumulating *E. coli* cells resulted in the formation of nostoxanthin and 2,2′-dihydroxy-β-carotene, respectively. Based on these results, a biochemical pathway for synthesis of nostoxanthin in *S. elodea* ATCC 31461 is proposed.

## Introduction

Carotenoids are isoprenoid pigments that are widely distributed in nature [1]. They can be synthesized by all known phototrophic organisms and by some non-phototrophic fungi, bacteria, and archaea [1], [2]. Due to their unique physiochemical properties, they have diverse biological functions in different organisms that either produce or consume them. These functions include their anticarcinogenic and antioxidant activity, protection against photo-oxidative damage, contribution to the light-harvesting process in photosynthesis, provitamin A property of β-carotene and as nutritional factors important for chronic disease prevention [3]–[5]. The interesting properties and beneficial effects on human health have drawn much attention. Over recent years, some identified carotenoids have been used as colorants, nutritional supplements and nutraceuticals for food, cosmetic and pharmaceutical purposes [6].

Carotenoid biosynthetic pathway has been extensively studied in various organisms and remarkable progress has been made. All carotenoids are derived from the isoprenoids pathway. The first step in the carotenoid biosynthetic pathway is the formation of geranylgeranyl pyrophosphate (GGPP) from farnesyl pyrophosphate (FPP) by GGPP synthase. Then two GGPP molecules are condensed head to head by phytoene synthase, resulting in the formation of the first carotene phytoene. After phytoene formation the biosynthetic pathways vary in different organisms resulting in a wide carotenoid diversity. In most bacteria, the colorless phytoene is desaturased by the phytoene desaturase through four consecutive steps to produce the red pigment lycopene. Various further modifications by cyclases, hydroxylases, ketolases and other enzymes lead to the formation of different carotenoids [7]–[9].

*Sphingomonas elodea* ATCC 31461 (originally designated as *Pseudomonas elodea*, also referred to as *Sphingomonas paucimobilis*) was isolated as a Gram-negative bacterium capable of producing gellan gum [10]. It synthesizes a yellow carotenoid identified as nostoxanthin ((2*R*,3*R*,2′*R*,3′*R*)-β,β-Carotene-2,3,2′,3′-tetrol) [11]. Nostoxanthin is a poly-hydroxy derivative of β-carotene isolated only from some prokaryotes, including some species of cyanobacteria [12], the novel bacteriochlorophyll a-containing bacterium *Sandarakinorhabdus limnophila* [13], the moderately thermophilic aerobic photosynthetic bacterium *Porphyrobacter tepidarius* [14], the marine bacterium *Brevundimonas* sp. strain SD212 [15], the strictly aerobic photosynthetic bacterium *Erythrobacter longus* [16], and most *Sphingomonas* species [11]. Although all the necessary genes required to synthesize nostoxanthin have been identified from *Brevundimonas* sp. strain SD212, *Brevundimonas vesicularis* strain DC263 and *Thermosynechococcus elongatus* strain BP-1 [12], [15], [17], genetic data on nostoxanthin biosynthesis are limited and the carotenoid biosynthetic pathway of *Sphingomonas* species remains unclear.

We previously cloned and identified the *crtI* gene encoding phytoene desaturase in *S. elodea* ATCC 31461 [18]. In the present study, we describe the cloning and characterization of the other genes involved in the nostoxanthin biosynthetic pathway of this organism. Using gene inactivation together with chromatographic and spectroscopic analysis of the pigments, we determined the functions of four carotenoid biosynthesis genes. In particular, the *crtG* gene encoding the 2,2′-β-hydroxylase, was also found in the carotenoid biosynthesis gene cluster of *S. elodea* ATCC 31461. Moreover, the functions of the two hydroxylase genes, *crtZ* and *crtG*, both responsible for hydroxylation of β-carotene were confirmed by complementation studies using *Escherichia coli* producing different carotenoids. As a result, the nostoxanthin biosynthetic pathway has been proposed.

## Results

### Cloning of the nostoxanthin biosynthetic genes

From SiteFinding-PCR [19], a 9.6-kb fragment containing a carotenoid biosynthesis gene cluster was obtained by assembly of the PCR products. However, the *crtZ* gene is not contained as a member of this cluster. Although we performed several rounds of SiteFinding-PCR (obtained about 22 kb sequence data), we could not amplify the *crtZ* gene. Because the *crtZ* gene is not linked to the carotenoid biosynthesis gene cluster, the CODEHOP strategy was used to generate PCR primers for partial *crtZ* fragment amplification. An internal *crtZ* fragment of 266 bp was isolated by PCR amplification using primers deduced from conserved internal domains of CrtZs of sphingomonadales (see Materials and Methods), providing sequence information for designing specific primers for SiteFinding-PCR that generated full-length *crtZ*. Sequences have been deposited in GenBank under accession number JN224892 for the carotenoid biosynthesis gene cluster and JN224893 for *crtZ*.

### Sequence analysis of the nostoxanthin biosynthetic genes

**Figure 1:**
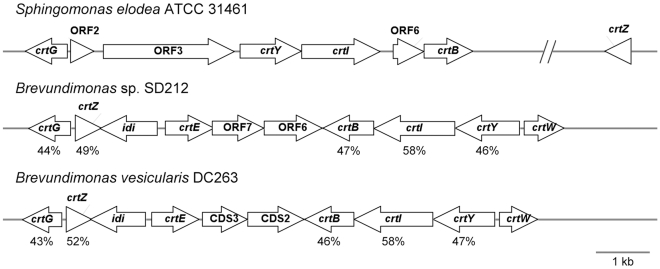
Comparison of the carotenoid biosynthetic genes for *S. elodea* ATCC 31461 and the *Brevundimonas* strains. The GenBank accession numbers are as follows: JN224892 and JN224893, *S. elodea* ATCC 31461; AB181388, *Brevundimonas* sp.SD212 [15]; DQ309446, *Brevundimonas vesicularis* DC263 [17]. The genes are presented as arrows pointing in the direction of their transcriptions. The percentage values below each gene designate the percent amino acid identity between the *S. elodea* ATCC 31461 nostoxanthin biosynthetic enzymes and the corresponding gene from the *Brevundimonas* strains.

The carotenoid biosynthesis gene cluster is 8,412 bp long and contains 7 putative ORFs. Based on the alignments of the deduced amino acid sequences with those of known carotenogenic enzymes, four of these ORFs were respectively identified as putative genes encoding carotenoid biosynthetic enzymes (Table 1). The translated amino acid sequence of ORF3 showed the highest sequence identity (55%) to *Caulobacter crescentus* CB15 TonB-dependent receptor. The deduced amino acid sequences of the other two ORFs (ORF2 and ORF6) exhibit no overall homology with any other known enzymes. These four carotenogenic genes have been tentatively assigned as *crtG*, *crtY*, *crtI* and *crtB*, encoding respectively putative 2,2′-β-hydroxylase, lycopene cyclase, phytoene desaturase and phytoene synthase. Similar to other *Sphingomons* genes, the carotenogenic genes displayed a high GC content (66% to 73%), and a high frequency of G or C in the position of codons [20]. The gene direction and the percent amino acid identity were compared among the carotenoid biosynthetic genes of *Brevundimonas* (Fig. 1). The individual ATCC 31461 carotenogenic gene products showed moderate identity to the corresponding proteins derived from *Brevundimonas*, ranging from 43% to 58%. Among the different carotenogenic genes, *crtI* appeared to be the most conserved gene. Compared with the organization of the carotenoid biosynthesis gene clusters isolated from other nostoxanthin-producing bacteria, this gene cluster organization was different. The *crtY*, *crtI*, and *crtB* were found to be in the same orientation, whereas the *crtG* gene preceded these three but was oriented in the opposite direction. The stop codon of the *crtY* gene is followed immediately by the start of *crtI* combined with a frameshift. Except these two genes, other carotenogenic genes are not closely physically linked.

**Table 1:** Homology analysis of the nostoxanthin biosynthetic genes in *S. elodea* ATCC 31461.

Gene	Length (bp)	Assigned gene product	The greatest sequence identity with other known carotenogenic enzyme (%)	GeneBank accession no.
*crtG*	798	2,2′-β-ionone ring hydroxylase	44, CrtG, *Brevundimonas aurantiaca*	DQ309446
*crtY*	1158	lycopene cyclase	44, CrtY, *Pantoea* sp. C1B1Y	AY876938
*crtI*	1479	phytoene desaturase	68, CrtI, *Erythrobacter longus*	D83514
*crtB*	924	phytoene synthase	53, CrtB, *Pantoea agglomerans*	M87280
*crtZ*	501	3,3′-β-ionone ring hydroxylase	54, CrtZ, *Pantoea ananatis*	D90087

### Carotenoid identification of knockout mutants

**Figure 2:**
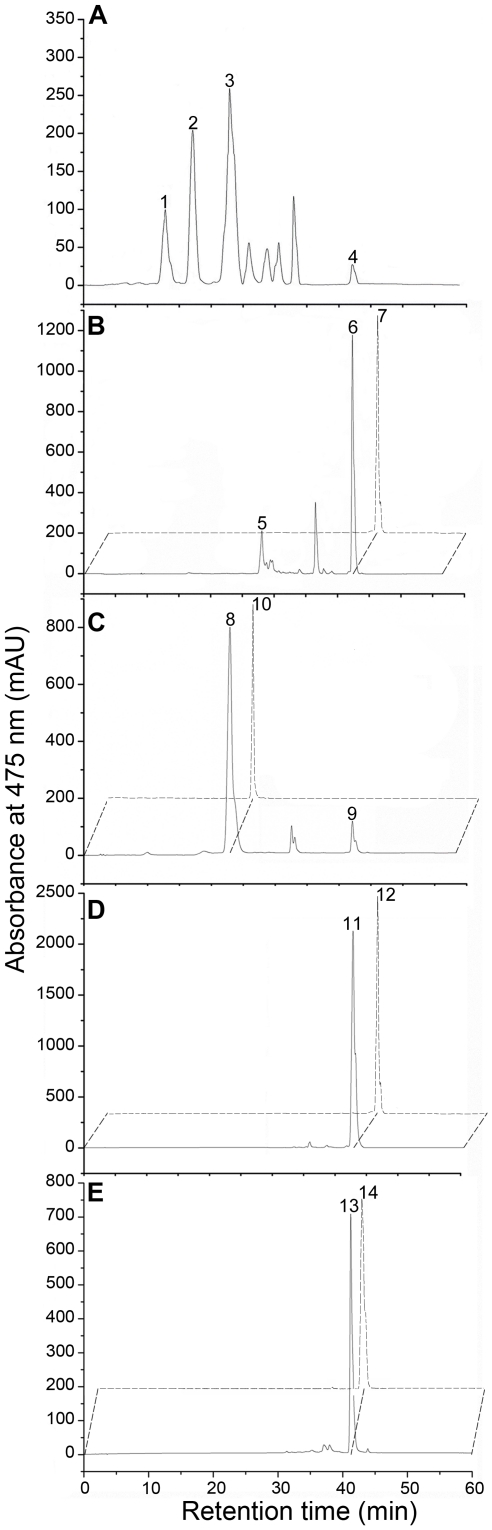
HPLC elution profiles of the pigments extracted from the wild-type and the knockout mutants. Solid lines, the wild-type and the knockout mutants; dashed lines, the control assays. A, wild-type; B, Δ*crtZ*; C, Δ*crtG*; D, Δ*crtZG*; E, Δ*crtY*. The major pigments were identified as nostoxanthin (peak 1, λ_max_: 274, 450, 476; [M+H]^+^: 601), caloxanthin (peak 2, λ_max_: 276, 450, 476; [M+H]^+^: 585), zeaxanthin (peaks 3, 8 and 10, λ_max_: 275, 450, 476; [M+H]^+^: 569), 2,2′-dihydroxy-β-carotene (peak 5, λ_max_: 272, 450, 476; [M+H]^+^: 569), β-carotene (peaks 4, 6, 7, 9,11 and 12, λ_max_:452, 476; [M+H]^+^: 537) and lycopene (peak 13 and 14, λ_max_:296, 362, 446, 470, 502; [M+H]^+^: 537).

HPLC analysis of the carotenoids isolated from the cells of ATCC 31461 showed several peaks at 475 nm. On the basis of previous studies as well as mass spectrometic analysis, peaks 1 through 4 were identified as nostoxanthin, caloxanthin, zeaxanthin and β-carotene, respectively (Fig. 2A). The other peaks might be impurities of the carotenoid extract.

Targeted deletions of the candidate carotenogenic genes in *S. elodea* ATCC 31461 were performed by double-crossover recombination. All knockout mutants were analyzed for carotenoid identification by LC-APCI-MS. Compared to the wild-type strain, Δ*crtZ* did not produce 3-hydroxy carotenoids, and the pigments were separated into three major peaks. Peaks 5 and 6 were determined to be 2, 2′-dihydroxy-β-carotene and β-carotene respectively (Fig. 2B). These results demonstrated that an inactive *crtZ* gene inhibited 3,3′-hydroxylation of β-carotene synthesis. In mutant Δ*crtG*, nostoxanthin and caloxanthin were not detected. The major pigment was identified as zeaxanthin (peak 8, Fig. 2C), and a small amount of its precursor β-carotene was present (peak 9, Fig. 2C). These data indicate that in this mutant the 2,2′-hydroxylation step was missing. The HPLC elution profile of the carotenoids accumulated by double knockout mutant Δ*crtZG* showed a single major peak. This peak correspond to non-hydroxylated β-carotene (peak 11, Fig. 2D), indicating that CrtZ and CrtG were responsible for hydroxylation of the β-ionone rings to produce nostoxanthin. The *crtY* knockout mutant exhibited a light red pigmentation, distinct from the yellow one of the wild-type strain. This mutant, called Δ*crtY*, accumulated lycopene that was absent from the wild-type strain (peak 13, Fig. 2E), suggesting that lycopene cyclization was impaired in this mutant. In addition, a knockout mutant of *crtI* accumulating phytoene instead of the final nostoxanthin has been described earlier [18]. Because phytoene is colorless, this mutant forms white colonies. Similarly, the colonies formed by the mutant Δ*crtB* were also white, which serve as a visible marker for screening. HPLC analysis monitored at 475 or 284 nm showed that the *crtB* knockout mutant did not produce any carotenoids, indicating that knockout of *crtB* blocked carotenoid synthesis.

In order to determine whether the two ORFs (ORF2 and ORF6) were involved in carotenoid biosynthesis, we further deleted these two ORFs respectively. Knockout of ORF2 or ORF6 had no effect on the formation of nostoxanthin, indicating that these two ORFs were not related to carotenoid biosynthesis.

### Functional complementation of CrtZ and CrtG in *E. coli*

**Figure 3:**
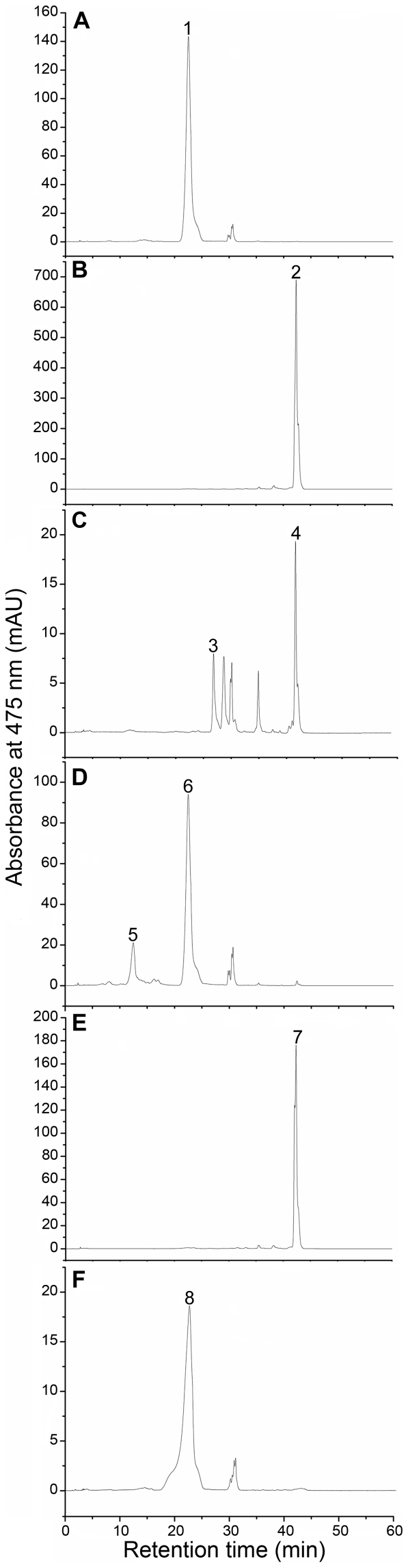
HPLC elution profiles of the pigments extracted from the *E. coli* JM109 transformants. A, pACCAR16ΔcrtX-Z; B, pACCAR16ΔcrtX; C, pACCAR16ΔcrtX and pUC18G; D, pACCAR16ΔcrtX-Z and pUC18G; E, pACCAR16ΔcrtX and pUC18; F, pACCAR16ΔcrtX-Z and pUC18. The pigments were identified as β-carotene (peaks 2, 4 and 7), 2,2′-dihydroxy-β-carotene (peak 3), zeaxanthin (peak 1, 6 and 8) and nostoxanthin (peak 5).

In order to further determine the function of CrtZ, plasmid pACCAR16ΔcrtX-Z was constructed and then introduced in *E. coli* JM109. The transformant formed bright yellow colonies on LB agar plates containing requisite antibiotics. HPLC analysis of the carotenoids extracted from the transformant indicated that zeaxanthin was the predominant carotenoid (peak 1, Fig. 3A). A control culture, with the pACCAR16ΔcrtX plasmid, contained only β-carotene (peak 2, Fig. 3B). Therefore, we confirmed that this gene encoded the β-carotene hydroxylase converting β-carotene to zeaxanthin. To confirm the biological role of CrtG and to identify the molecular mechanism for β-carotene 2,2′-hydroxylation, we expressed the gene in zeaxanthin and β-carotene-accumulating *E. coli* cells and analyzed the carotenoid content by HPLC. The zeaxanthin and β-carotene-accumulating *E. coli* transformats to be used in this study was made by the introduction of plasmid pACCAR16ΔcrtX-Z or pACCAR16ΔcrtX in strain JM109. These two plasmids contain the p15A replication origin which is compatible with pUC18, making them amenable to replicate in conjunction with pUC18G within the same cell [21]. When plasmid pUC18G was introduced into the β-carotene-accumulating *E. coli* transformant, the new transformant accumulated 2,2′-dihydroxy-β-carotene (peak 3, Fig. 3C). In the transformant carrying pACCAR16ΔcrtX-Z and pUC18G, CrtG catalyzed the formation of nostoxanthin (peak 5, Fig. 3D). As the negative controls, *E. coli* cotransformed with pUC18 plus either pACCAR16ΔcrtX or pACCAR16ΔcrtX-Z was used, resulting in the accumulation of β-carotene or zeaxanthin (peak 7, Fig. 3E; peak 8, Fig. 3F). These results show that *crtG* encoding 2,2′-dihydroxy-β-carotene hydroxylase, was capable of hydroxylating β-carotene and zeaxanthin.

## Discussion

**Figure 4:**
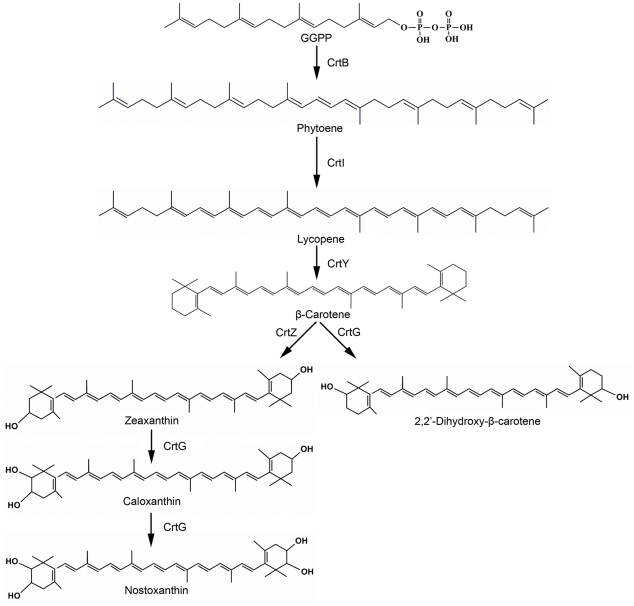
The pathway of carotenoid biosynthesis in *S. elodea* ATCC 31461. The gene products responsible for each enzymatic reaction are indicated.

Bacterial strains of the genus *Sphingomonas* are often isolated from contaminated environments [22], and most *Sphingomonas* species produce nostoxanthin [11]. The precise function of this unique carotenoid in these bacteria is unclear, likely it is associated with tolerance to environmental stress due to the antioxidant activity of carotenoids. In this study, the carotenogenic genes involved in the nostoxanthin biosynthetic pathway of *S. elodea* ATCC 31461 have been cloned and identified. Based on our results, we propose a biochemical pathway for synthesis of nostoxanthin in *S. elodea* ATCC 31461 (Fig. 4). The CrtG enzyme was first found in *Brevundimonas* species and the homologs of this hydroxylase was later found in cyanobacteria [12], [15]. The carotenoid biosynthetic genes in cyanobacteria are dispersed throughout the whole genome, while in typical bacteria, these genes are often clustered into large operons [23]. In the *Brevundimonas* carotenoid gene clusters, the *crtG* and *crtZ* genes were found to be adjacent to each other but oriented in the opposite direction [15], [17]. However, the *crtZ* gene of ATCC 31461 was not linked to *crtG* but located outside the carotenoid biosynthesis gene cluster. The genes *crtI* and *crtB* have been isolated from a variety of organisms. These two genes were often closely physically linked, whereas the *crtI* and *crtB* in the cluster of ATCC 31461 were separated by an unknown gene (ORF6). The relatively loose organization of these carotenogenic genes might have occurred through a rare event during evolution.

**Figure 5:**
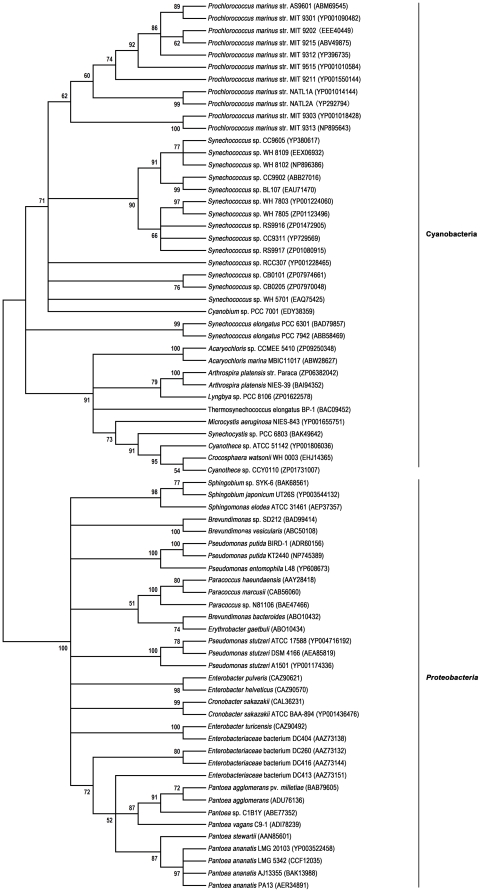
Neighbor-joining tree based on the β-carotene hydroxylase sequences. Bootstrap values expressed as percentage of 1000 replications are shown next to each node (values below 50% are not shown). The GenBank accession numbers are given in parentheses.

Various *crtZ* genes have been isolated from α-*proteobacteria*, γ-*proteobacteria*, eubacteria and cyanobacteria. A molecular phylogenetic tree was established based on the β-carotene hydroxylase sequences (Fig. 5). According to the phylogenetic tree, the β-carotene hydroxylases from the *Sphingomonadaceae* family constituted a group independent from the others, and ATCC 31461 CrtZ formed a distinct lineage within this group, indicating that ATCC 31461 CrtZ was in an independent phylogenetic position from the other genera.

The *crtG* gene from *Brevundimonas* sp. SD212 has been reported to exhibit undetectable activity on β-carotene [15]. In contrast, ATCC 31461 CrtG could convert β-carotene into 2,2′-dihydroxy-β-carotene. This result was consistent with that of the CrtG from *Brevundimonas aurantiaca* [17]. Similar to CrtGs from *Brevundimonas* species, CrtG from *S. elodea* ATCC 31461 also exhibits intriguingly partial homology with the middle region of animal sterol-C5-desaturase involved in cholesterol biosynthesis. BLAST was used to search the GenBank database with the ATCC 31461 CrtG protein sequence as query. We found CrtG homologs, which have several annotations, depending on the source genome, as encoding sterol desaturases, fatty acid hydroxylases, or C-5 sterol desaturases. The ATCC 31461 CrtG shares a larger degree of sequence identity (76%–78%) with the homologs from the *Sphingomonadaceae* family, suggesting these homologs may play the similar role in carotenoid-producing bacteria of this family.

*S. elodea* ATCC 31461 is used for biotechnological production of the commercial exopolysaccharide gellan gum. Gellan gum has a wide range of applications in food, pharmaceutical, and other industries due to its excellent rheological characteristics and unique structure [18], [24]. However, its costly downstream purification process limits its economic viability. To reduce process costs, several colorless mutants have been isolated by random mutagenesis and gene knockout [18], [25]. The knowledge about the biosynthesis of nostoxanthin in this bacterium can be used to obtain colorless mutants to further test for gellan production. Heterologous carotenoid production in *E. coli* or higher plants is a powerful method for the biosynthesis of natural carotenoids of interest [26]. In particular, the 2-hydroxy and 2,2′-dihydroxy carotenoids which are difficult to synthesis chemically have improved antioxidant activity [15]. The *crtG* gene identified here can be used for heterologous production of these novel carotenoids through combinatorial biosynthesis.

## Materials and Methods

### Bacterial strains and culture conditions

Table 2 gives the bacteria strains and plasmids used in this study. Except otherwise indicated, the wild-type and mutant strains of *S. elodea* ATCC 31461 were cultured in the yeast extract-peptone-glucose (YPG) medium (0.3% yeast extract, 0.5% peptone and 2% glucose, w/v) at 30°C for 72 h and stored at 4°C. *E. coli* S17-1 and *E. coli* DH5α were the hosts for genetic manipulation whereas *E. coli* JM109 was used either for production of carotenoids or complementation experiments. *E. coli* strains were cultured aerobically in Luria-Bertani (LB) medium at 37°C or 30°C. The solid medium contained agar (15 g/L). For carotenoid production by *Sphingomonas* strains, inoculum was developed by transferring one loop full of the organism from slant culture to the YPG medium in 500 mL Erlenmeyer flasks. The flasks were incubated on a rotary shaker at 200 rpm and 30°C for 24 h for inoculum development. Then 10 mL broth was used as inoculum for 100 mL of yeast extract-glucose (YG) medium (0.05% yeast extract, 1% glucose, 0.015% K_2_HPO_4_, 0.01% NaHPO_4_, 0.01% MgSO_4_·7H_2_O, w/v) in 500 mL Erlenmeyer flasks and incubated on a rotary shaker at 200 rpm and 30°C for 48 h. *E. coli* strains for pigment analysis were grown in LB medium at 37°C over night, of which 6 mL was used to inoculate 100 mL LB medium in 500 mL Erlenmeyer flasks and incubated at 30°C for 2 days. Isopropyl-β-D-thiogalactopyranoside (IPTG) was used at 1 mM for induction of the culture as required. When necessary, antibiotics were used at the following concentrations (µg/mL): streptomycin (25), kanamycin (50), ampicillin (15 for *Sphingomonas* and 100 for *E. coli*), chloramphenicol (25), and tetracycline (5 for *Sphingomonas* and 25 for *E. coli*).

**Table 2:** Bacterial strains and plasmids used in this study.

Strain or plasmid	Relevant properties or derivation	Source
Strain		
*Sphingomonas elodea*		
ATCC 31461	Wild type	ATCC
Δ*crtB*	*crtB* knockout mutant of ATCC 31461	This study
Δ*crtY*	*crtY* knockout mutant of ATCC 31461	This study
Δ*crtZ*	*crtZ* knockout mutant of ATCC 31461	This study
Δ*crtG*	*crtG* knockout mutant of ATCC 31461	This study
Δ*crtZG*	*crtZ-crtG* double knockout mutant of ATCC 31461	This study
*Escherichia coli*		
DH5*α*	F^−^, φ80d*lac*ZΔM15, Δ(*lacZYA-argF*)U169, *deoR*, *recA*1, *endA*1, *hsdR*17(r_k_^−^,m_k_^+^), *phoA*, *supE*44, λ^−^, *thi*-1, *gyrA*96, *relA*1	Lab collection
S17-1	*rec*A *pro hsd*R RP4-2-Tc::Mu-Km::Tn7; mobilizer strain	[37]
JM109	*recA1 endA1 gyrA96 thi hsdR17 supE44 relA1*Δ(lac-proAB)/F′[*proAB^+^lacI^q^ lacZ*ΔM15]	Lab collection
HB101/pRK2013	HB101 harbouring pRK2013, Km^r^	[30]
Plasmid		
pRK2013	ColE1 *mob*+*tra*_RK2_Δ*rep*_RK2_ *repE* ^−^ Km^r^	[30]
pLO3	Tc^r^ *sacB*, RP4 *oriT*, ColE1 *ori*	[29]
pLO3B	pLO3 carrying upstream 581 bp and downstream 667 bp of *crtB*	This study
pLO3Y	pLO3 carrying upstream 482 bp and downstream 470 bp of *crtY*	This study
pLO3Z	pLO3 carrying upstream 545 bp and downstream 513 bp of *crtZ*	This study
pLO3G	pLO3 carrying upstream 644 bp and downstream 406 bp of *crtG*	This study
pACCAR16*ΔcrtX*	Cm^r^, plasmid carrying the *crtE*, *crtB*, *crtI*, and *crtY* genes from *Pantoea ananatis*	[21]
pACCAR16*ΔcrtX-Z*	Cm^r^, plasmid carrying the *crtE*, *crtB*, *crtI*, *crtY* genes from *Pantoea ananatis* and *crtZ* from ATCC 31461	This study
pMD19-T	PCR cloning vector, Amp^r^	Takara
pUC18	High-copy-number expression vector, Amp^r^	Takara
pUC18G	pUC18 carrying the *crtG* gene from ATCC 31461	This study

### Recombinant DNA techniques

Plasmid preparations, PCR reactions, transformations and other standard molecular biology techniques were carried out as described elsewhere [27] or as instructed by suppliers. The restriction enzymes, DNA polymerase and DNA ligation kit were purchased from Takara (Dalian, China).

### Cloning of the carotenoid biosynthesis genes

The phytoene desaturase gene of *S. elodea* ATCC 31461 was previously cloned and identified [18]. To clone other carotenoid biosynthesis genes from this organism, the sequences obtained previously were extended using the SiteFinding-PCR method [19]. From SiteFinding-PCR, a carotenoid biosynthesis gene cluster was obtained by merging the sequences of different DNA fragments. The CODEHOP (Consensus-degenerate hybrid oligonucleotide primers) strategy (http://blocks.fhcrc.org/blocks/codehop.html) was used to generate PCR primers for partial *crtZ* fragment amplification [28]. Primer design was based on a created set of blocks that represented highly conserved regions of homologous proteins. Specific blocks were made using the Block Maker program (http://blocks.fhcrc.org/blocks/make_blocks.html) by submitting eight bacterial β-carotene hydroxylase sequences: ABO10434 from *Erythrobacter gaetbuli*, ABC62608 from *Erythrobacter litoralis* HTCC 2594, EAQ28084 from *Erythrobacter* sp. NAP1, BAD 99406 from *Brevundimonas* sp. SD 212, ABO10432 from *Brevundimonas bacteroides*, ABC50108 from *Brevundimonas vesicularis*, EAS50417 from *Aurantimonas manganoxydans* SI85-9A1, and ABS69756 from *Xanthobacter autotrophicus* Py2. Several sets of primers were designed by the CODEHOP program, using the default parameters of the CODEHOP server. Primers *crtZ*sense and *crtZ*antisense were selected to amplify the expected sequences. The 3′-terminal and 5′-terminal sequences of *crtZ* were also acquired by SiteFinding-PCR method. Specific primers used for SiteFinding-PCR were designed according to the 3′-end and 5′-end sequences. The primers used for cloning of the biosynthesis genes are shown in Table S1. All PCR fragments were cloned using the pMD19-T vector for sequencing. To reveal any mispairing and sequencing error, the genes were cloned from genomic DNA by PCR with Pyrobest DNA polymerase, an enzyme with high fidelity (Takara, Dalian, China). These PCR fragments were subsequently cloned and characterized by DNA sequencing.

### Comparative sequence analysis and phylogenetic tree construction

Database searches were run with the BLAST server at the National Center for Biotechnology Information (http://www.ncbi.nlm.nih.gov/BLAST). Multiple sequence alignments were performed with the ClustalW program at the European Molecular Biology Laboratory (http://www.ebi.ac.uk/clustalw). Subsequent adjustments of these alignments were done manually. A phylogenetic tree was constructed by the neighbor-joining (NJ) method using MEGA version 4.0 from ClustalW alignment. Bootstrap support was estimated using 1000 replicates for distance analysis.

### Construction of knockout mutants

The candidate carotenoid biosynthetic genes and ORFs were inactivated by double-crossover homologous recombination. The upstream and downstream flanking sequences of genes selected for knockout were amplified by PCR using genomic DNA templates isolated from wild-type *S. elodea* ATCC 31461 with the primers described in Table S2. The primers used to amplify the flank fragments of the target genes contained SacI, XbaI, or PstI restriction sites. PCR products of respective gene were digested with the appropriate restriction enzymes, ligated to a suicide vector pLO3 [29], and used to transform *E. coli* S17-1.

Transfer of the respective recombinant plasmids (pLO3B, pLO3Y, plO3Z, and pLO3G) to *S. elodea* was performed by triparental filter mating using *E. coli* HB101/pRK2013 as the help strain [30], [31]. *E. coli* S17-1 containing the recombinant plasmid was used as a conjugal donor, whereas pRK2013 can mobilize and promote conjugal transfer. The protocol for triparental filter mating was performed as described elsewhere [32]. Transconjugants were selected on YM plates containing tetracycline at 5 µg/mL and subsequent YPG plates containing 8% sucrose. The knockout mutants were isolated by selecting for lost of sucrose toxicity encoded by the *sacB* gene of pLO3 [33] and antibiotic susceptibility, followed by PCR screening using the verification primers for those with the correct excision and DNA sequencing.

### Construction of plasmids for expression of crtZ and crtG in *E. coli*

The candidate β-carotene hydroxylase (CrtZ) and 2,2′-β-hydroxylase (CrtG) enzymes were assayed by expressing the genes in *E. coli* JM109 engineered to accumulate different carotenoid substrates. Plasmid for expression of *crtZ* (pACCAR16ΔcrtX-Z) was constructed as follows. The candidate *crtZ* gene was amplified by PCR with the primers AvaI-*crtZ*-sense and HindIII-*crtZ*-anti, which were designed to contain AvaI and HindIII restriction sites, respectively. The *Pantoea ananatis crtE* gene was amplified from plasmid pACCAR16ΔcrtX by PCR with the primers HindIII-*crtE*-sense and SalI-*crtE*-anti, which were designed to contain HindIII and SalI restriction sites, respectively. The plasmid pACCAR16ΔcrtX carrying the carotenoid biosynthetic genes responsible for synthesis of β-carotene (*crtE*, *crtB*, *crtI*, and *crtY* of *pantoea ananatis*) was kindly provided by Dr. Norihiko Misawa [34]. The above PCR products were digested with the appropriate restriction enzymes, and plasmid pACCAR16ΔcrtX was excised with AvaI and SalI. Then the two PCR fragments were ligated with the larger fragment of pACCAR16ΔcrtX carrying *P. ananatis crtB*, *crtI*, and *crtY*, to give plasmid pACCAR16ΔcrtX-Z.

For expression of candidate *crtG* in *E. coli*, plasmid pUC18G was constructed. Primers EcoRI-*crtG*-sense and BamHI-*crtG*-anti were used to amplify the candidate *crtG* gene with engineered EcoRI and BamHI restriction sites, and cloned into the EcoRI/BamHI site of pUC18 vector to create the expression vector pUC18G. The expressed CrtG protein was fused to a lead sequence of β-galactosidase under the control of the *lac* promoter by pUC18 [15]. Primers used for heterologous expression of *crtZ* and *crtG* in *E. coli* are listed in Table S3.

### Isolation and identification of carotenoids

Cells grown as described above were harvested by centrifugation (6,000× *g* at 4°C for 15 min) and washed three times with sterilized water. The pigments were extracted from the cells with acetone/methanol (2∶1, v/v) under N_2_ at 4°C and centrifuged to collect the supernatant. The extraction was repeated three times in dark to ensure all pigments were transferred to the liquid phase. Subsequently, the extracts were dried under vacuum and then saponified as described elsewhere [11]. After drying, the pigment was stored under N_2_ at −80°C until further analysis. The saponified extracts were resuspended in 1 mL choloform/2-propanol (1∶1, v/v). Carotenoids were identified by HPLC using an Agilent 1200 high performance liquid chromatography (Agilent, USA) equipped with a diode-array detector and a Hypersil ODS-C18 column (4.6 mm×250 mm, 5 µm). Pigments were eluted with 85% methanol/5% 2-propanol/10% water for the first 20 min, then a linear gradient to 70% methanol/30% 2-propanol within 30 min, and maintained these final conditions for 10 min. The flow rate was 1 mL/min. HPLC-purified compounds were analyzed by LC-MS with an Agilent 1200 series LC/MSD Trap SL mass spectrometer system. The system control and data acquisition were performed using LC/MSD Trap Software 4.2 (Bruker). The MS parameters were set as follows: ion source, atmospheric pressure chemical ionisation (APCI); nebulisher, 60 psi; drying gas, nitrogen; drying gas temperature, 350°C; drying gas flow, 5.0 L/min; APCI temperature, 350°C; HV capillary, 3500 V; and scan range, 100 to 2200 *m*/*z*. Carotenoids were identified by retention time, features of absorption spectra and mass spectra in comparison to standard compounds or with reported data [11], [12], [15]–[17], [35], [36]. The standards of β-carotene, zeaxanthin and lycopene were obtained from CaroteNature (Lupsingen, Switzerland).

## Supporting Information

Table S1 **DNA oligonucleotide primers used for cloning.**
(DOC)

Table S2 **Primers designs for gene knockout constructs.** Nucleotides which are in bold show changes that were made in the sequence to engineer restriction sites for cloning. Restriction sites are underlined.
(DOC)

Table S3 **Primers used for heterologous expression of** ***crtZ*** **and** ***crtG*** **in** ***E. coli*** **.** Nucleotides which are in bold show changes that were made in the sequence to engineer restriction sites for cloning. Restriction sites are underlined.
(DOC)
