# Supplementary material for: Cloning and Characterization of Genes Involved in Nostoxanthin Biosynthesis of Sphingomonas elodea ATCC 31461
Source: PLoS One. 2012 Apr 11;7(4):e35099. doi: 10.1371/journal.pone.0035099 (PMC3324416; doi:10.1371/journal.pone.0035099)
Supplement: Table S1 — DNA oligonucleotide primers used for cloning. (DOC) [file pone.0035099.s001.doc]

**Table S1.** DNA oligonucleotide primers used for cloning.

| Name | Sequences (5′ to 3′) |
| --- | --- |
| CODEHOP primers used for partial *crtZ* fragment amplification | |
| *crtZ*sense | GCCTGGTCGATGCACAAGTAYRTNATGCAYG |
| *crtZ*antisense | CGGCGTGGTGCAGCYKRTGNGCYTG |
| SiteFinders (SiteF1-SiteF4) and their primers (SFP1 and SFP2) used for SiteFinding-PCR | |
| SiteF1 | CACGACACGCTACTCAACACACCACCACGCACAGCGTCCTCAANNNNNNCATGG |
| SiteF2 | CACGACACGCTACTCAACACACCACCACGCACAGCGTCCTCAANNNNNNCATGC |
| SiteF3 | CACGACACGCTACTCAACACACCACCACGCACAGCGTCCTCAANNNNNNGCCT |
| SiteF4 | CACGACACGCTACTCAACACACCACCACGCACAGCGTCCTCAANNNNNNGCCACG |
| SFP1 | CACGACACGCTACTCAACAC |
| SFP2 | ACTCAACACACCACCACGCACAGC |
