# Supplementary material for: Cloning and Characterization of Genes Involved in Nostoxanthin Biosynthesis of Sphingomonas elodea ATCC 31461
Source: PLoS One. 2012 Apr 11;7(4):e35099. doi: 10.1371/journal.pone.0035099 (PMC3324416; doi:10.1371/journal.pone.0035099)
Supplement: Table S2 — Primers designs for gene knockout constructs. Nucleotides which are in bold show changes that were made in the sequence to engineer restriction sites for cloning. Restriction sites are underlined. (DOC) [file pone.0035099.s002.doc]

**Table S2.** Primers designs for gene knockout constructs. Nucleotides which are in bold show changes that were made in the sequence to engineer restriction sites for cloning. Restriction sites are underlined.

| Gene | Direction | Strand | Sequences (5′ to 3′) |
| --- | --- | --- | --- |
| *crtB* | Upstream | Sense | **GTGAGCTC**CGTATCGCCGTCTATTGC (SacI site underlined) |
|  |  | Antisense | **GCGTCTAGA**GCGCATTACAGCTGTTCG (XbaI site underlined) |
|  | Downstream | Sense | **ATTCTAGA**CGCTGACGCTCAGCCGTT  (XbaI site underlined) |
|  |  | Antisense | **ACCTGCAG**CTGGTTTTCCGCATACTC  (PstI site underlined) |
|  | Verification | Sense | CGGCGAAAACACGCTATCA |
|  |  | Antisense | GCTGAACGGCTGAGCGTC |
| *crtY* | Upstream | Sense | **AAGAGCTC**ACCGCCAAGATCACCCAG  (SacI site underlined) |
|  |  | Antisense | **GCATCTAGA**AGGCATGACTGCCGCCTA  (XbaI site underlined) |
|  | Downstream | Sense | **TATCTAGA**CTTTCCGCGATCTGGAGC  (XbaI site underlined) |
|  |  | Antisense | **TACTGCAG**CGACTTGAAGTCCAGAAA  (PstI site underlined) |
|  | Verification | Sense | GGCAGTGTCCGCTACGAT |
|  |  | Antisense | GCTTCACATAGCCTTCCTCATA |
| *crtZ* | Upstream | Sense | **GACGAGCTC**ATCGATCCCGGCTATTAT  (SacI site underlined) |
|  |  | Antisense | **GGCTCTAGA**CAGCAAAAAGGCGTTGAG  (XbaI site underlined) |
|  | Downstream | Sense | **TGTCTAGA**CCGGATTGAGGGCCATCC  (XbaI site underlined) |
|  |  | Antisense | **TGCTGCAG**GTGGTCGATTACAGGCTC  (PstI site underlined) |
|  | Verification | sense | CGCGCCTGCCGGAACTGA |
|  |  | antisense | CGTGGAACTGCTCGGGGGAG |
| *crtG* | Upstream | Sense | **GCTGAGCTC**GTTGATGAAGGGAGTCTA  (SacI site underlined) |
|  |  | Antisense | **TATCTAGA**GTTCATGCGCCGATCTGC  (XbaI site underlined) |
|  | Downstream | Sense | **GCATCTAGA**GCTGGAGCTTGATTTCACC  (XbaI site underlined) |
|  |  | Antisense | **TACTGCAG**CAGACGATCAGAAACCCC  (PstI site underlined) |
|  | Verification | Sense | TGGCGACCACTCCCAACAG |
|  |  | Antisense | CGGAATGCCCATGAAGGTG |
