# Supplementary material for: Cloning and Characterization of Genes Involved in Nostoxanthin Biosynthesis of Sphingomonas elodea ATCC 31461
Source: PLoS One. 2012 Apr 11;7(4):e35099. doi: 10.1371/journal.pone.0035099 (PMC3324416; doi:10.1371/journal.pone.0035099)
Supplement: Table S3 — Primers used for heterologous expression of crtZ and crtG in E. coli . Nucleotides which are in bold show changes that were made in the sequence to engineer restriction sites for cloning. Restriction sites are underlined. (DOC) [file pone.0035099.s003.doc]

**Table S3.** Primers used for heterologous expression of *crtZ* and *crtG* in *E. coli*.

Nucleotides which are in bold show changes that were made in the sequence to engineer restriction sites for cloning. Restriction sites are underlined.

| Name | | Sequences (5′ to 3′) | |
| --- | --- | --- | --- |
| AvaI-*crtZ*-sense | **TACCCGAG**AAGGAGGCTAGATATGTCGCCGCTCAACGCCTTTTTG (AvaI site underlined) | |  |
| HindIII-*crt*Z-anti | | **ACAAGCTT**TCAATGATGATGATGATGATGATCCGGGATGGTGATCCG (HindIII site underlined) | |
| HindIII-*crtE*-sense | | **ACAAGCTT**TTATAAGGACAGCCCGAATG  (HindIII site underlined) | |
| SalI-*crtE*-anti | | **CAGTCGAC**ATCCTTAACTGACGGCAG  (SalI site underlined) | |
| EcoRI-*crtG*-sense | | **AAGAATTC**GATGAACGTCGCGCTTGCC  (EcoRI site underlined) | |
| BamHI-*crtG*-anti | | **CTGAGGATCC**AATCAAGCTCCAGCGTCG  (BamHI site underlined) | |
